# Effect of diets with different energy concentrations on growth performance, carcass characteristics and meat chemical composition of broiler chickens in dry tropics

**DOI:** 10.1186/s40064-016-3608-0

**Published:** 2016-11-09

**Authors:** F. Infante-Rodríguez, J. Salinas-Chavira, M. F. Montaño-Gómez, O. M. Manríquez-Nuñez, V. M. González-Vizcarra, O. F. Guevara-Florentino, J. A. Ramírez De León

**Affiliations:** 1 Facultad de Medicina Veterinaria y Zootecnia, Universidad Autónoma de Tamaulipas, Km 5.5 Carretera Victoria, Mante, C.P. 87000 Ciudad Victoria, Tamaulipas Mexico; 2 Instituto de Investigaciones en Ciencias Veterinarias, Universidad Autónoma de Baja California, Mexicali, Baja California Mexico

**Keywords:** Energy level, Growth performance, Carcass, Meat composition, Broiler chicken

## Abstract

**Background:**

Diets with increasing levels of energy were fed for 42 days to 200, 1-day old male broiler chickens to evaluate growth performance, carcass characteristics and chemical composition of meat. The study was performed in the subtropical area of northeastern Mexico. Treatments diets (T) for starter and finisher phases had apparent metabolizable energy (AME; kcal/kg) of: 2960 and 3040 (T1); 3000 and 3080 (T2); 3040 and 3120 (T3); 3080 and 3160 (T4), respectively. Within each of the growing phases the four treatment diets were formulated to contain similar levels of crude protein, amino acids, and other nutrients. In a completely randomized design, birds were allocated to the four treatments with five replicates (floor pens) of 10 birds each. The trial was divided in two phases (starter and finisher) of 21 days each (42 days total).

**Results:**

Weight gain was not influenced by energy level; however, feed conversion efficiency was improved in the diets with 3040 and 3120 kcal/kg AME (T3; P < 0.05). There was no influence of treatment on total carcass weight or carcass cuts (P > 0.05). Meat from breast muscle had similar crude protein percentages among treatments; ether extract was higher in T1 than T4 (P < 0.05). The percentages of water, ether extract, ash and crude protein in thigh meat were not significantly different (P > 0.05) among treatments.

**Conclusions:**

For this study carried out in a dry tropical area, the moderate increase in dietary energy concentration (diet with 3040 and 3120 kcal/kg AME, T3) enhanced feed conversion efficiency of broiler chickens.

## Background

Poultry constituted 63% of animal production in Mexico: 33.5% was contributed by production of broiler chickens, almost 29% by egg production and 0.1% by production of turkeys (Unión Nacional de Avicultores (UNA) 2015). In Mexico, the cost of feed plus the cost of the chicks represents approximately 90% of broiler production costs (Unión Nacional de Avicultores (UNA) 2015). Diets for broilers are based on grains such as sorghum or corn plus soybean meal, which have been expensive in recent years. In this sense, improvements in feed efficiency reduce production cost in broiler chicken production (Branson and Hernández 2012).

Broiler meat is an important protein source for consumers. In Mexico, per capita consumption in 2014 was 29.3 kg of broiler chicken meat. Low and medium income households prefer “dark meat” (broiler hindquarters), while consumers with higher incomes prefer cuts of “white meat” (breast) and other value-added chicken products (Branson and Hernández 2012). A greater yield of cuts of higher market value (breast) is desirable. In addition, consumers prefer meat with lower fat content mainly due to health concerns. There are some reports on the influence of nutrient concentration in diet on growth performance or carcass yield; however, there is limited information of influence on meat (breast and thigh + drumstick) chemical composition, in particular for broiler chicken production under tropical conditions.

Increasing the energy and protein density of diets for broiler chickens has given inconclusive results. A preliminary study (Waldroup et al. 1990) found no effect of energy concentration of diets on growth performance or abdominal fat, although higher energy density increased dressing percentage in females, but not in males. Similarly, others found no effect of dietary energy level on carcass yield or abdominal fat (Nunes et al. 2012; Duarte et al. 2014). In contrast, Marcu et al. (2012a) reported improved growth performance and carcass yield for the main cuts of broiler chickens fed diets with high energy and protein levels. Marcu et al. (2013) found that increasing dietary energy and protein elevated breast weight and muscle mass, and reduced fat content, while reducing nutrient levels decreased protein content and elevated fat content in pectoral muscle. In contrast, Ferreira et al. (2015) produced meat with less fat in broiler chickens fed low energy diets, although growth was decreased. Several factors could have influenced the results of growth performance, carcass yield of cuts or meat chemical composition of broiler chickens fed diets with different nutrient density; one of them was genotype of broiler chickens. Kim et al. (2012) reported different responses to energy concentration with different strains of broiler chickens.

The continuous improvement in the genetics of broiler chickens has enhanced productive and carcass characteristics, including carcass leanness. Research on broiler nutrition and feeding must also continue to synergize with improvements in genetic potential. This research is also important in areas where the climatic conditions may influence the productive performance, carcass yield and meat chemical composition. In northeastern Mexico the standard diets for starter and finisher phases for broiler chickens contain 2994 and 3081 kcal/kg ME, respectively. An energy increase in the diets to 3013 and 3111 kcal/kg ME for starter and finisher phases improved growth performance of broiler chickens (Orduña-Hernández et al. 2016); although they did not explore higher energy levels and did not study carcass characteristics. The objective of this research was to assess productive performance, carcass yield of major cuts and chemical composition of meat from breast and thigh of broiler chicks in a 42-days feeding trial in the subtropical area of northeastern Mexico.

## Methods

### Description of the study area

This study was carried out at the Facultad de Medicina Veterinaria y Zootecnia, Universidad Autónoma de Tamaulipas, in Ciudad Victoria, Tamaulipas (subtropical area in northeastern Mexico). The area is located at 23°44′06″N and 97°09′50″W, at an altitude of 340 m. The mean annual rainfall is 900 mm, and the average temperature is 25 °C (INEGI. Instituto Nacional de Estadística, Geografía e Informática 2006). These climatic characteristics are typical for dry tropics. During the experiment average minimum and maximum temperatures averaged 23.2 and 33.6 °C, respectively.

### Management and feeding of experimental broiler chickens

All procedures involving animal care and management were in accordance with and approved by the Bioethics Committee of the Facultad de Medicina Veterinaria y Zootecnia, Universidad Autónoma de Tamaulipas.

Two hundred, 1-day-old male Ross broiler chickens weighing 42 g on average were obtained from a commercial hatchery. Each treatment (or diet) included 50 birds randomly assigned with five replications of ten animals each. During the entire experiment, birds were housed in 20 floor pens with wood shavings as litter. Twenty-four hours of light per day were provided during the entire trial. Each pen had an automatic drinker and a feeder filled manually. Space allocation was at 10 birds per square meter. Water and feed were offered ad libitum. Birds were vaccinated on day 7 of the trial against fowlpox (by wing puncture) and against Newcastle (ocular) using the La Sota strain.

The chickens were raised following standard commercial practice. Two feeding phases were used: 1–21 and 22–42 days of age, for starter and finisher phases, respectively. There were four treatments (T). For starter and finisher the apparent metabolizable energy (AME) levels were: 2960 and 3040 (T1); 3000 and 3080 (T2); 3040 and 3120 (T3); 3080 and 3160 kcal/kg. Dietary AME was increased by augmenting vegetable oil in the diet, with small adjustments to the amounts of sorghum grain and soybean meal to keep the levels of CP and other nutrients the same across diets. All diets were prepared according to National Research Council (NRC) (1994) poultry recommendations and are shown in Tables 1 and 2.

**Table 1:** Experimental diets for the starter phase (% as-fed basis)

	T1	T2	T3	T4
Ingredient				
Sorghum, grain	60.9	59.9	58.9	57.8
Soybean meal	33.3	33.5	33.7	33.9
Vegetable oil	1.8	2.6	3.4	4.2
Premix^a^	4.0	4.0	4.0	4.0
Total	100	100	100	100
Calculated analysis				
AME, kcal/kg	2960	3000	3040	3080
CP (%)	21.4	21.4	21.4	21.4
Lysine (%)	1.1	1.1	1.1	1.1
Methionine (%)	0.5	0.5	0.5	0.5
Ca (%)	1.0	1.0	1.0	1.0
P (%)	0.7	0.7	0.7	0.7

^a^Concentrated premix for starter phase broiler chickens. Contained: calcium mono-ortho phosphate, calcium carbonate, salt, growth promoter (BMD and 3-nitro), coccidiostat (sodium monensin), mineral oil, ethoxyquin, vitamin A acetate, vitamin D_3_, vitamin E acetate, vitamin K3, riboflavin (B2), vitamin B_12_, niacin, d-calcium pantotenate, choline chloride, butylated hydroxytoluene (BHT). Premix calculated to contain: calcium, 21.40%; total phosphorus, 8.10%; sodium, 3.40%; l-lysine hydrochloride, 0.80%; dl-methionine, 4.15%

**Table 2:** Experimental diets for the finisher phase (% as-fed basis)

	T1	T2	T3	T4
Ingredient				
Sorghum, grain	67.6	66.6	65.6	64.6
Soybean meal	26	26.2	26.4	26.6
Vegetable oil	2.1	2.9	3.7	4.5
Premix^a^	4	4	4	4
Pigment	0.3	0.3	0.3	0.3
Total	100	100	100	100
Calculated analysis				
AME (kcal/kg)	3040	3080	3120	3160
CP (%)	18.7	18.7	18.7	18.7
Lysine (%)	1.0	1.0	1.0	1.0
Methionine (%)	0.5	0.5	0.5	0.5
Ca (%)	0.9	0.9	0.9	0.9
P (%)	0.7	0.7	0.7	0.7

^a^Concentrated premix for finisher phase broiler chickens. Contained: calcium mono-ortho phosphate, calcium carbonate, salt, growth promoter (BMD and 3-nitro), coccidiostat (sodium monensin), mineral oil, ethoxyquin, vitamin A acetate, vitamin D_3_, vitamin E acetate, vitamin K3, riboflavin (B2), vitamin B_12_, niacin, calcium d-pantotenate, choline chloride, BHT. Premix calculated to contain: calcium, 19.80%; total phosphorus, 7.30%; sodium, 3.70%; l-lysine hydrochloride, 4.33%; dl-methionine, 5.15%

During the feeding trial, body weight and feed intake were measured weekly and feed conversion efficiency (feed intake/weight gain) was calculated. At the end of the trial, two birds per cage, selected at random, were sacrificed by cervical dislocation (Mexican official law NOM 033-ZOO-1995) for carcass determinations and chemical composition of breast and thigh meat. Carcass weight without viscera was used to estimate hot carcass yield. Then, the carcass was dissected for main cuts: breast, thighs plus drumsticks, wings, and back; back fat was also weighed. A sample of left side of breast and thigh from each carcass was obtained and frozen in nylon bags until laboratory analyses were performed. Muscle samples were analyzed for dry matter (oven at 105 °C for 24 h), ash (furnace at 550 °C for 3 h), ether extract and crude protein percentages (AOAC 1990).

### Statistical analyses

A completely randomized design with 4 treatments and 5 replicates was used in the statistical analysis. The treatments evaluated were the dietary energy levels. For growth performance (weight gain, feed intake and feed conversion efficiency) the replicate was the average for all broiler chickens in each pen. For carcass evaluation and meat chemical composition the replicate was the average of two birds (selected at random) per pen. An analysis of variance with Tukey’s test for mean comparisons was applied. Statistical significance was declared at P < 0.05. For statistical analyses the GLM procedures of SAS (2007) were used.

## Results

### Growth performance

Broiler chicken productive performance is shown in Table 3. For the starter phase, there was no effect (P > 0.05) of dietary energy concentration on weight gain. Broiler chickens in T3 had lower feed intake and better feed conversion efficiency than those in T1 and T2 (P < 0.05). For the finisher phase, there was no effect (P > 0.05) of energy level on weight gain. Broilers in T3 had lower feed intake than those in T2. Also broilers in T3 had better feed conversion efficiency than those in T1 and T2 (P < 0.05). For the 42-day feeding period, there was no treatment effect (P > 0.05) on weight. Broiler chickens in T3 had lower feed intake than those in T1 and T2 and showed better feed conversion efficiency than those in T1, T2 and T4 (P < 0.05).

**Table 3:** Performance measures of broiler chickens (cumulative average per bird)

	T1	T2	T3	T4	SEM
Starter phase (1–21 days)					
Weight gain (g)	750	764	766	740	18.74
Feed intake (g)	1207^a^	1224^a^	1080^b^	1124^ab^	36.10
Feed conversion efficiency	1.61^a^	1.61^a^	1.41^b^	1.52^ab^	0.05
Finisher phase (22–42 days)					
Weight gain (g)	1723	1757	1844	1719	46.94
Feed intake (g)	3556^ab^	3634^a^	3417^b^	3432^ab^	68.85
Feed conversion efficiency	2.07^a^	2.08^a^	1.85^b^	2.00^ab^	0.05
Total trial (1–42 days)					
Weight gain (g)	2473	2520	2609	2459	57.35
Feed intake (g)	4763^ab^	4858^a^	4497^c^	4556^bc^	76.10
Feed conversion efficiency	1.93^a^	1.93^a^	1.73^b^	1.86^a^	0.04

^abc^Different superscripts in a row differ (P < 0.05)

### Carcass characteristics

Results of carcass characteristics of broilers chickens are shown in Table 4. There was no influence of treatment on weight of carcass, breast, drumstick plus thighs, wings and back fat (P > 0.05). Animals fed T3 and T4 had greater (P < 0.05) back weight than those in T1. Carcass yield was similar among treatments (P > 0.05).

**Table 4:** Carcass characteristics of broiler chickens

	T1	T2	T3	T4	SEM
Live weight (g)	2781	2812	2879	2852	72.95
Hot carcass weight (g)	1956	2012	2068	2046	58.66
Breast weight (g)	724	745	762	744	26.72
Thigh + drumstick weight (g)	604	608	628	627	16.14
Wings weight (g)	221	218	221	225	6.18
Back weight (g)	355^b^	382^ab^	396^a^	412^a^	12.62
Back fat weight (g)	28.50	29.50	34.00	31.30	2.97
Carcass yield (%)	70.32	71.57	71.75	71.76	0.63

^ab^Different superscripts in a row differ (P < 0.05)

### Proximate composition of breast and thigh meat

Results of chemical composition of broiler chicken breast and thigh meat are shown in Table 5. Breast muscle had similar (P > 0.05) crude protein percentage among treatments. Water percentage was higher in T3 than T4; ash content was higher in T3 than T2; ether extract was higher in breast muscle of birds in T1 than those in T4 (P < 0.05). Thigh muscle had similar (P > 0.05) water, ether extract, ash and crude protein percentages among treatments (P > 0.05).

**Table 5:** Chemical composition of breast and thigh muscles (wet-basis)

	T1	T2	T3	T4	SEM
Breast					
Water (%)	74.17^ab^	74.48^ab^	75.09^a^	74.11^b^	0.31
Dry matter (%)	25.83^ab^	25.52^ab^	24.91^b^	25.89^a^	0.31
Ash (%)	3.95^ab^	3.69^b^	4.28^a^	4.02^ab^	0.11
Ether extract (%)	1.85^a^	1.15^b^	1.50^ab^	1.22^b^	0.20
Crude protein (%)	23.88	23.93	22.62	23.62	0.52
Thigh					
Water (%)	77.46	77.25	77.66	77.08	0.43
Dry matter (%)	22.54	22.75	22.34	22.92	0.43
Ash (%)	1.08	1.04	1.20	1.22	0.06
Ether extract (%)	2.13	2.64	2.73	2.67	0.26
Crude protein (%)	19.47	19.20	19.60	19.81	0.33

^ab^Different superscripts in a row differ (P < 0.05)

## Discussion

### Growth performance

Carbohydrates in grains are the main source of energy in broilers diets; however, lipids are included in these diets to cover the energy requirements of birds for maximum growth performance (National Research Council (NRC) 1994). In the current study the increase in dietary energy concentration was achieved by increasing the amount of vegetable oil. Energy concentration did not influence broiler chicken weight gain; however, feed conversion was improved using diet T3, which maintained weight gain with lower feed intake. Other studies are in agreement with this finding (Jafarnejad and Sedegh 2011; Ferreira et al. 2015; Orduña-Hernández et al. 2016). In different reports on broiler chickens, there is no clear indication about the effects of energy level in diet on weight gain, feed intake or feed efficiency. In studies using diets similar to those of the current study, Tancharoenrat and Ravindran (2014) observed that an increase in energy level improved weight gain and feed conversion with no effect on feed intake, while Kim et al. (2012) observed reduced feed intake with higher dietary energy. In studies with energy levels lower than those used in the present study, higher dietary ME improved broiler growth performance (Aftab 2009; Ullah et al. 2012).

For the present study, energy level in diet did not influence broilers’ weight; however, feed intake was reduced. In contrast, Houshmand et al. (2011) found that broiler chickens fed low energy diets were heavier than those on the standard diet. Tooci et al. (2009) compared concentrate diets (3010, 3150 and 3200 kcal/kg ME respectively for starter, grower and finisher phases) versus diluted diets (2800 kcal/kg ME) and reported that feed intake of broiler chickens was not influenced by energy content in diets, but concentrate diets improved weight gain and feed conversion. In addition to energy, amino acid levels in diet also influenced growth performance of broiler chickens. Zhai et al. (2014) reported that low energy diet with high amino acid levels depressed broilers’ feed intake and weight gain. Broiler chickens fed low energy diet with low amino acids had similar growth performance to those in high energy diets.

### Carcass characteristics

In the current study dietary energy level did not influence processed carcass weight, breast, drumstick + thighs, wings and back fat weight or carcass yields. Others have reported similar responses in broiler chickens fed different energy levels (Nunes et al. 2012; Kim et al. 2012). In contrast, higher energy and protein diets increased yield of breast (Marcu et al. 2013), weight of carcass and yields of breast and thigh muscle; whereas drumsticks, wings and other carcass components (head, neck, back and legs) were reduced (Marcu et al. 2012b). Marcu et al. (2012a) also showed that diets for broilers having extra energy and protein improved carcass yield in females, but not in males. For both genders, breast and drumstick were improved by extra energy and protein; thigh was not influenced by energy level, but the high energy reduced yield of wings in females. Starter, grower and finisher diets had 3026, 3142 and 3196 kcal/kg of ME for the control group; while for the extra energy group, the respective values of diets were 3281, 3439 and 3483 kcal/kg of ME. Zhao et al. (2008) found that carcass, breast, thigh and abdominal fat were increased with higher dietary energy and lysine. The optimum growth and carcass performance were obtained at a dietary energy of 3300 versus 3099 kcal/kg ME; the levels of dietary lysine were 1.2% from 0 to 3 weeks of age and 1.1% from 4 to 6 weeks of age. Rosa et al. (2007) used diets with 2950, 3200 and 3400 kcal/kg ME, but found no effect on carcass yield, breast or back fat, although the increase in energy concentration depressed yield of thigh + drumstick and increased abdominal fat. Also Wang et al. (2014) found that broiler chickens fed extra energy and amino acids levels, showed increased abdominal fat, while weight of the carcass cuts were not affected. Milošević et al. (2013) reported that increased energy density with continuous illumination increased abdominal fat.

### Proximate composition of breast and thigh meat

In the present study the increase in energy level of the diet had no effect on crude protein percentage in breast muscle, whereas T1 treatment had more lipids in meat from breast than T4. Thigh lipid percentage was not influenced by energy level and protein percentage was greater in T3 than T2. Marcu et al. (2012b) reported that reducing dietary energy level lowered crude protein and increased lipid content of broiler breast and thigh muscle. Marcu et al. (2013) fed broiler diets with 2950, 3100 or 3250 kcal/kg ME, and reported quadratic effects for lipid and crude protein content of breast that were maximized at 3100 kcal/kg ME and were reduced when dietary energy was lower or higher. These results suggest that an increase in dietary energy level for broiler chickens may not increase meat lipids in pectoral muscle.

Ferreira et al. (2015) found different effects and reported lower intramuscular fat content in meat from birds fed diets with lower energy level. Rosa et al. (2007) reported that carcass chemical composition changes in different genetic groups; however, for commercial Ross 308 broilers there was reduced crude protein and increased lipid in carcass with increased dietary energy level in broilers. Others found no effect of dietary energy level on chemical composition of carcass muscle of broilers (Corduk et al. 2007; Gómez-Rosales et al. 2012).

## Conclusions

Body weight gain was not influenced by energy level in diets for broiler chickens; however, there was improved feed conversion efficiency in treatment 3, where 3040 and 3120 kcal/kg AME were fed in the starter and finisher diets, respectively. Weights of carcass and main cuts (breast, thigh + drumsticks and wings) were not influenced by dietary energy level. For breast muscle, crude protein percentage was not influenced by dietary treatments, although lipid percentage was higher when feeding the low energy diets (T1) compared to the T4 high energy diets. For thigh muscle, lipid or crude protein percentages were not influenced by dietary treatments. In this study conducted in a subtropical area, the moderate increase in dietary energy concentration (diet T3) enhanced feed conversion efficiency of broiler chickens.

## Abbreviations

T: treatments
AME: apparent metabolizable energy
ME: metabolizable energy
CP: crude protein
Ca: calcium
P: phosphorus
